# Interaction Effects of Farm-Scale Management of Natural Enemy Resources and the Surrounding Seminatural Habitat on Insect Biological Control

**DOI:** 10.3390/insects16030286

**Published:** 2025-03-10

**Authors:** Blas Lavandero, Enrique Maldonado-Santos, Estefania Muñoz-Quilodran, Mauricio González-Chang, Francisca Zepeda-Paulo, Ángel Salazar-Rojas, Cinthya Villegas

**Affiliations:** 1 Laboratorio de Control Biológico, Instituto de Ciencias Biológicas, Universidad de Talca, 2 Norte 685, Talca 3460000, Chile; enrique.maldonado@utalca.cl (E.M.-S.); emunoz16@alumnos.utalca.cl (E.M.-Q.); fzepedapaulo@gmail.com (F.Z.-P.); cinthyavillegasg@gmail.com (C.V.); 2 Instituto de Producción y Sanidad Vegetal, Facultad de Ciencias Agrarias y Alimentarias, Universidad Austral de Chile, Valdivia 5091000, Chile; mauricio.gonzalez.chang@uach.cl; 3 Facultad de Ciencias Agrarias y Forestales, Universidad Católica del Maule, Talca 3340000, Chile; angel@agroecosistemas.cl; 4 Programa Doctorado Medio Ambiente y Sociedad UPO 4, Universidad Pablo de Olavide (UPO 4), 41013 Sevilla, Spain

**Keywords:** *Diaeretiella rapae*, *Brevicoryne brassicae*, seminatural habitats, flower strips, nectar provision

## Abstract

Agricultural land use and its disruption of natural landscapes threaten the provision of ecosystem services, such as biological control by natural enemies, because of habitat simplification and management intensification. However, most studies that evaluate the effect of local management practices rarely identify and include other important predictors such as landscape compositional values. We studied the effect of adding flower strips at farms on the control of aphids in a seminatural habitat (SNH) gradient. We found significantly less aphids on farms with flower strips and more SNH at the second sampling date as well as a greater proportion of mummies with flowers early in the season, with a greater proportion of mummies at a greater %SNH at the end of the season. Foraging predators responded to the %SNH of farms without flowers only on the second sampling date, which coincided with their highest mean abundances. Our data suggests that aphid parasitism was enhanced by flowers, having a potential effect early in the season, which ultimately explained the reduction in aphid numbers thereafter. On the other hand, the effects perceived on predator abundances seemed to be more date- and landscape-sensitive. Flower strips of faba beans and buckwheat in the field as well as the %SNH surrounding farms positively affected *Diaeretiella rapae* and could therefore be an important management strategy to decrease *Brevicoryne brassicae* densities in brassica crops.

## Simple Summary

The disruption of natural landscapes threatens the provision of ecosystem services, such as biological control. The surrounding non-crop habitat as well as local management practices may improve the biological control of crop pests. We studied the effect of adding flower strips on the control of aphids taking into account the effect of the surrounding landscape of 14 farms. We found significantly less aphids on farms with flower strips that had more surrounding non-crop habitats, which also had a greater proportion of mummies (parasitoids) early in the season under these same conditions. Predators, in contrast to parasitoids, responded more to the surrounding non-crop habitats later in the season, which coincided with their highest mean abundances. Our data suggests that aphid parasitism was enhanced by flowers, having a potential effect early in the season, which ultimately could explain the reduction in aphids thereafter. On the other hand, the effects perceived on general predator abundances seemed to be more related to their abundance and surrounding non-crop habitats. Flower strips of faba beans and buckwheat in the fields of farms with enough surrounding non-crop vegetation could therefore be an important management strategy to decrease brassica crop aphids in the field.

## 1. Introduction

Agricultural land use and its disruption of natural landscapes threaten the provision of ecosystem services, such as biological control by natural enemies, due to habitat simplification and management intensification. The notion that surrounding non-crop habitat may improve the biological control of crop pests by beneficial natural enemies (e.g., predators and parasitoids) has emerged as a paradigm for conservation biocontrol [1]. In this regard, research on the impacts of land management (conserving or restoring habitats) on pest control has focused on both the farm and landscape scales (habitat management within farms and the surrounding landscape, respectively). From a landscape perspective, complexity can improve biocontrol through increased natural enemy abundance and pest predation/parasitization rates [1], but effective pest suppression may also depend on other context-related variables, such as local management [2]. On the other hand, farm-scale management practices such as flower strips, beetle banks, or inter-row vegetation (e.g., intercropping) have proven to bolster enemy abundance and richness, contributing to increased pest control [3]; however, there is little information on how these interact within the landscape context. Currently, agroecosystem diversification strategies and sustainable agricultural practices at local and landscape scales are increasingly encouraged by entities such as the FAO, with the aim of enhancing the ecological interactions and synergies that promote the functioning and supply of ecosystem services and reduce external inputs to agriculture. Moreover, local farming practices such as habitat management are particularly seen by farmers as promising practices to bolster the biological control of pests [4]. However, much remains to be learned about how local habitat management is modulated by landscape effects and agricultural practices at different spatial and temporal scales [5,6], which makes its influence on natural enemy biology uncertain as well as site-specific and thus its implementation difficult. Indeed, interactions between ecological processes and agricultural practices are influenced not only by habitat management at the farm-scale (high in-field plant diversification) but also the surrounding habitat structure at the landscape level as well as farming practices (conventional or organic farming) [7]. The enhancement in plant biodiversity, through the enrichment of the farm matrix, could increase the abundance and/or diversity of natural enemies by creating complex habitats that provide shelter (overwinter and insecticide applications), nectar, alternative hosts/preys, and pollen (SNAP: the major resources provided by plants to natural enemies [3]), but the extent to which it can subsidize direct suppressive effects on pests needs to be measured across different spatial and landscape scales. In particular, plant nectar (floral and extra floral) is an important sugar source for parasitoid wasps, and its availability can impact the longevity, fecundity, as well the ability of parasitoids to find and attack hosts [8]. Restoring flowering plants to field crops that provide significant resources to natural enemies is a key component of conservation biological control [9,10]. Therefore, the extent to which habitat diversification strategies can subsidize biological control needs to consider the landscape contexts of these studies and crop types. The elements surrounding farms such as field margins, hedgerows, woody and herbaceous habitats and other crops may provide shelter, alternative hosts, and sugar sources. On the other hand, simple landscapes, with poor non-crop habitat resources, which do not provide shelter, alternative hosts, or sugar sources, may not limit the effect of natural enemies if management practices supplement sugar resources by providing floral nectar through the use of flower rows, intercropping, or honeydew directly from the hosts of these parasitoids [11]. Therefore, to understand the extent to which the promotion of augmenting resource subsidies for natural enemies (floral resources, honeydew, shelter, and host/preys) affects pest control, the landscape context should always be taken into account.

We studied the impact of a single aspect of farm management, the implementation of flower strips, considering the percentage of seminatural and natural elements of each of the farms as a continuous linear predictor. At each brassica farm in central Chile, we measured the abundance of generalist predators and specialist natural enemies (parasitoids), and its effect on the biological control of the main aphid pest. We hypothesized that as seminatural and natural elements increase in agricultural landscapes, the effect of local nectar provision through flower strips diminishes.

## 2. Materials and Methods

**Figure 1:**
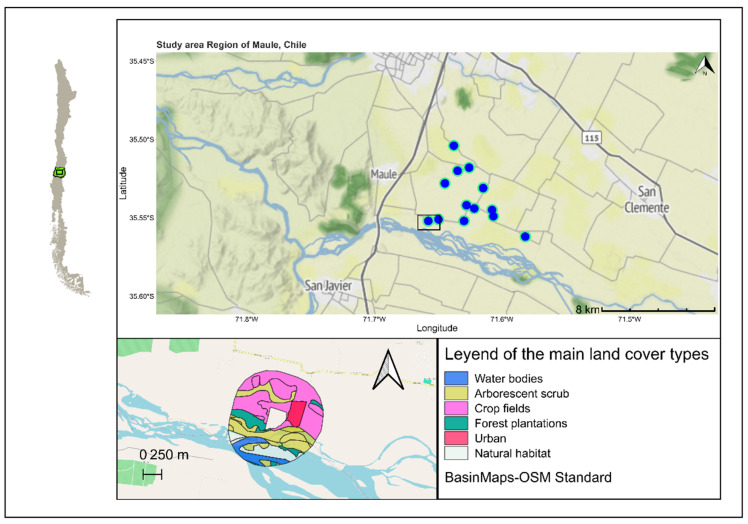
Geographical representation of the farms used for this study in the central valley of Chile with coordinates. Each farm represented by a blue dot. In the bottom left, at scale, an example of land cover representation in a farm buffer (1 km diameter); see Table 1 for details.

A total of 20 farms were first selected in a landscape gradient (see Figure 1) during the spring of 2023. At half of these farms, 50 cm wide and 20 m long flower strips were established in autumn (*V. faba*) and again in spring (*F. esculentum*) to ensure a continuous supply of nectar, allowing similar levels of landscape context gradient for both management treatments (Figure 1). In this study, we focused on the main Aphidiinae parasitoids, the main aphids attacking brassica farms, and their most abundant predators. We previously evaluated the impact of several frequently used non-crop plant species in habitat management, *Fagopyrum esculentum* (Polygonaceae), *Borago officinalis P* (Boraginaceae), *Lobularia maritima* (Brassicaceae), *Coriandrum sativum* (Apiaceae), *Calendula officinalis* (Asteraceae), and *Vicia faba* (Fabaceae) [12], on the resource availability for parasitoids (floral and extra floral resources), quantifying insect visits, phenology, and ease of use in two agricultural landscapes of central Chile. For this pilot study, we selected the two most attractive and easy to use flower strips, which were *F. esculentum* and *V. faba* (unpublished data).

The central valley of Chile is characterized by a temperate Mediterranean climate, with dry summers and mild, rainy winters [13]. Temperatures vary from 25 to 35 °C in spring–summer (September–March) and between 3 and 13 °C in winter, with precipitation ranging from 22 to 130 mm during spring and from 300 to 900 mm in winter (June–August) [14,15]. The natural vegetation is characterized by sclerophyllous forest in the coastal mountain range and foothill of the Andes, thorn scrub (*Acacia caven*) mainly in the eastern part, with *Nothofagus* species forests mostly close to the Andes. Between these two mountains ranges, most agriculture is carried out; however, patches of natural and seminatural elements persist throughout [16]. In the last 40 years, annual field crop coverage has begun to decrease, and the coverage of export-oriented orchards and vineyards, as well as forest plantations, has increased (with a consequent 12.7–27.0% reduction per 10 years of native forests) [17].

### 2.1. Landscape Attributes and Site Selection

In order to compare the landscape attributes of farms with and without flower strips, the compositional complexity of the landscapes in the surrounding 1 km diameter circular buffer (Figure 1) was determined. In each buffer for each farm, using satellite images with the aid of QGIS software [18], all elements and percentages of cover using Chile map of landscape covers [16,19] were identified using supervised classification methods. All the information gathered was also complemented by field visits. The following covers were obtained: AWV: area without vegetation (ha); ST: urban settlement (ha); NF: native forests (ha); BW: water body (ha); AS: arborescent scrub (ha); PL: forest plantation (ha); GR: agricultural grassland (ha); CP: crop field (ha); TBF: total buffer (ha); BSH: natural habitat (ha); PBSH: proportion natural habitat (NF + AS); PBW: proportion of water bodies (BW); %SNH: proportion of seminatural area (PBSH + PBW). The percentage of seminatural area (%SNH) was determined as the sum of the following covers: natural forest, natural shrubs, and water bodies (as many natural and naturalized plants are associated with these areas). Pastures (GR) were not included in the % of SNH as, in the study area, managed exotic pastures did not present significant floral resources at the time of the experiment (winter to early spring). All selected farms were at least 1 ha in size, with the greatest experimental units not surpassing 2 continuous ha of brassicae crops. During the winter of 2023, historical rainfall occurred in central Chile (five- to ten-fold more than the average), which meant that many farmers lost their farms to river flooding [20]. Due to this, we were left with eight farms with flower strips and eight farms without flowers. However, one of the farmers decided to remove the brassica crop before we could perform measurements (the SNH for this farm was 24%), and we were left with one farm in the “with flower” treatment, with a SNH of 34%). The farms’ main crops included five farms with broccoli, five with cabbage, and four cauliflower farms. Analyses were run with and without all farms, without any significant variation in the general models (see Table S1), but, to ensure a more representative, parsimonious, and less biased comparison, we present only the results for six farms with flowers and eight farms without flowers, therefore achieving an SNH gradient of 0.5 to 10.83%. In this way the mean %SNH between the two groups was not significantly different with a mean ± SD of 3.62 ± 3.36 and 4.93 ± 2.77 for the flower and non-flower treatments, respectively (more details in Table 1). The percentage of arable crops in the buffer was highly correlated (t = −55.631, df = 1238, *p*-value < 2.2 × 10^−16^; cor −0.845) with the %SNH; therefore, farms with a higher SNH had a lower % of arable crops. Therefore, farms with a greater %SNH had less agricultural features as well (Table 1).

### 2.2. Abundance of Aphids and Parasitism Rates

Within the selected farms, two 50 m sampling transects were established 5 m from the edge (or flower strip); from these, 40 plants were inspected in a random pattern during four consecutive days to sample all farms. This sampling of aphid predators and parasitoids occurred at the peak of the aphid season, determined a priori from several years of sampling and growers’ information, just before the harvest, to reduce the chances of pesticide use by farmers. We sampled at two dates during this period of maximum density with a 30-day interval between the first (15 November 2023) and the second (15 December 2023) sampling periods (allowing for a total of 80 randomly selected plants per farm). At each sampling event, the total number of aphids was counted on the underside of 10 leaves per plant. The number of parasitoid mummies per plant was also registered. The presence of predators during the sampling was also registered per plant. A subsample per farm was collected and taken back to the laboratory to estimate species composition (aphids and parasitoids). All species were determined using keys from [21,22,23,24].

### 2.3. Abundance of Predators

For each group of farms, we also determined the abundance of predators via manually collecting, as well as with the help of an entomological aspirator through a standardized inspection, with a 5 min sampling effort per plant using the same 40 plants per farm (were aphids and mummies were counted) for both sampling dates. Additionally, ten pitfall traps per farm were randomly placed among the crop and collected after one week during the first sampling date. The organisms were then preserved in 70% ethanol for further identification to order or family.

### 2.4. Statistical Analyses

All analyses were carried out using R version 4.3.2. [25]. Data were first explored with measures of central tendency for mean, standard error, and standard deviation using the tidyverse package [26]. To understand the effect of the management treatment (main explanatory variable) in our study on predator abundance, aphid abundance, and parasitism rates, we used generalized linear mixed models with the lme4 package [27], assuming a negative binomial distribution for the count variables and binomial for the proportions (mummification rates). Random variables “farm” and nested “plant per farm” were included in the first full exploratory model. Random variables “farm” and “sample” effects were retained in all models as they presented a more parsimonious explication of the fixed variables based on the residual variance and the compared AIC values between models, without overdispersion (Table S1). We assumed a random intercept for the random factors, as exploratory analysis only indicated variation in the intercept per farm, following the recommendations in [28,29]. Landscape complexity measured as %SNH was used as a continuous predictor within all models. Model simplification from full models resulted in the removal of the triple interaction for most response variables, as the interaction between %SNH, treatment, and sampling date did not significantly decrease the amount of residual deviance of the model or significantly increase predictive power (only date:treatment and date: %SNH interactions were significant), with the exception of total predators, where the triple interaction described above was significant. Model overdispersion was analyzed through the aods3 package [30] to reduce the unexplained variability within the models. As he models under Poisson distribution were overdispersed, the final models assumed a negative binomial distribution of the response variables, which fixed the overdispersion in all selected models [29]. The resulting models were compared with a subsequent type II ANOVA to select the best model. The proportion of mummies per colony sample was analyzed considering a binomial distribution with a logit link function, and no overdispersion was detected. Model selection parameters can be seen in detail in Table S1.

## 3. Results

**Figure 2:**
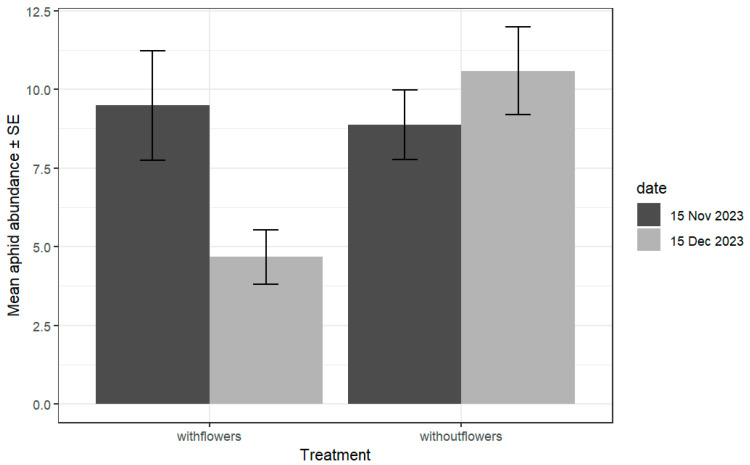
Mean number of aphids ± SE per plant at farms with and without flower strips, independent of the surrounding %SNA, for two sampling dates, f1 and f2. Significantly less aphids were found at farms with flowers during the second sampling date (date:treatment: χ^2^ = 20.53, df = 1, *p* = 5.8 × 10^−6^). Only significant differences were found between the 15 November and 15 December samplings and for farms with flowers according to Tukey’s test accounting for multiplicity.

**Figure 3:**
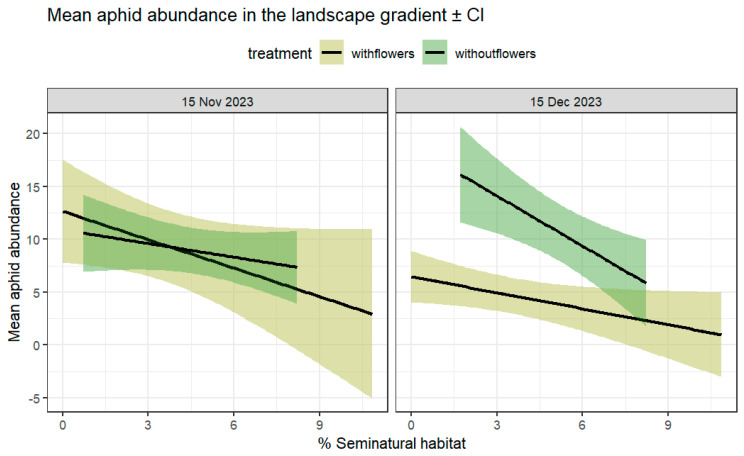
Mean number of aphids per plant in farms with and without flower strips (%SNH surrounding farms in a 1 km buffer as a continuous linear predictor). Lines represent means across samples ± 95% CI at two different sampling periods, 15 November (left) and 15 December (right), with and without flowers.

The mean number of aphids per plant varied among the farms, with a significant interaction between the date and the treatment, as well as for the interaction between %SNH and the date (Table 2). Significantly more aphids were observed at farms without flower strips during the second sampling event (date:treatment χ^2^ = 20.54; df = 1; *p* < 0.001) (Figure 2). The %SNH negatively affected the mean number of aphids per farm (during the second sampling date (SNH: date, χ^2^ = 25.89; df = 1; *p* < 0.001), with a significant aphid reduction at farms with flowers compared to farms without flowers during the same sampling period (Figure 2 and Figure 3). During the first sampling period, no significant differences were found among treatments. Only three aphid species were observed, with *Brevicoryne brassicae* and *Myzus persicae* being more than 80% of the sample. The mean proportion per species in the sample was 36.6% ± 0.38 for *M. persicae* and 45.28% ± 0.3 for *B. brassicae*; a small number of aphids corresponded to *A. gosippi* as well as unidentified nymphs.

The main parasitoid species that emerged from the mummies were *Diaeretiella rapae,* followed by *Praon volucre*, *Aphidius platensis,* and one individual of *A. matricariae*. From these, 92 *Diatarellela rape* parasitoids emerged from *B. brassicae* and 22 from *M. persicae*. Parasitoid richness per aphid species was the greatest in *B. brassicae* (all four species), with no significant differences between treatments (χ^2^ = 0.143, df = 1, *p* = 0.701). From these, the mean female sex ratio (female/female + male) ± SE for the emerged *D. rapae* per treatment was 0.42 ± 0.19 for farms with flowers and 0.37 ± 0.1 for farms without flowers.

**Figure 4:**
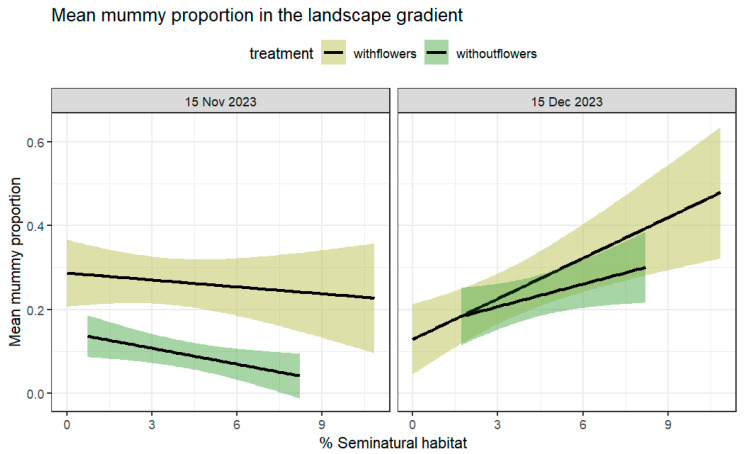
Mean proportion of mummies per plant at farms with and without flower strips as explained by the %SNH. The mean proportion of mummies significantly increased as the %SNH increased (SNH: χ^2^ = 11.76, df = 1, *p* = 0.0006; treatment: χ^2^ = 8.47, df = 1, *p* = 0.003). Dates: (left) 15 November, (right) 15 December.

The proportion of mummies per plant had a significant interaction between date and treatment, as well as for the %SNH and date (Table 2). Contrary to the aphids, there were significant differences for the first sampling date, where farms with flowers had a higher proportion of mummies at the first sampling date. There was a greater effect of the %SNH at the second sampling date, however without differences between treatments (Table 2, Figure 4). We did not find a significant difference in parasitoid richness between treatments (χ^2^ = 0.13; df = 1; *p* = 0.71), %SNH (χ^2^ = 0.0001; df = 1; *p* = 0.99), or aphid species (χ^2^ = 2.91; df = 4; *p* = 0.57).

**Figure 5:**
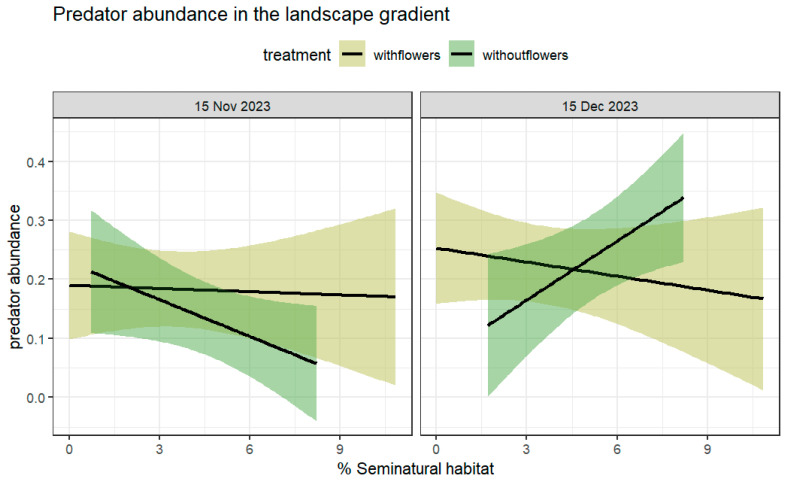
Mean number of predators foraging per plant (±95% CI) at farms with and without flower strips, %SNH in a surrounding 1 km buffer as a linear predictor, on two sampling dates: (left) 15 November, (right) 15 December. Abundance was higher at farms without flowers on the second sampling date when compared to the first sampling, which was affected by a higher %SNH (SNH:date:treatment χ^2^ = 10.14; df = 1; *p* < 0.01). Variation in slopes between the two sampling dates were significant (with flowers: %SNH: date:f2: estimator = 0.37; std. error = 0.11; z value = 3.18; *p* = 0.001) (see Table 2).

The total number predators collected foraging was 135 specimens (mean/plant ± SE: with flowers = 0.20 ± 0.551; without flowers = 0.18 ± 0.60) and consisted of Araneae (*n* = 28, mean/plant ± SE: with flowers = 1.14 ± 0.06; *n* = 32, without flowers = 1.28 ± 0.16) and Coccinelidae (*n* = 37, mean/plant ± SE: with flowers = 1.04 ± 0.21; *n* = 38, without flowers = 1.29 ± 0.55). We did not observe a significant increase in abundance at farms with flower strips compared to farms without flowers (χ^2^ = 0.44; df = 1; *p* = 0.51). The %SNH surrounding each farm was not significant (χ^2^ = 0.0016; df = 1; *p* = 0.97), but predator abundance differed within both dates (χ^2^ = 4.26; df = 1; *p* < 0.05). The %SHN did not interact with the presence of flower strips (χ^2^ = 0.005; df = 1; *p* = 0.94). The sampling date did not interact with the %SNH (χ^2^ = 3.49; df = 1; *p* = 0.062) or the presence of flower strips (χ^2^ = 0.08; df = 1; *p* = 0.78). However, the sampling date interacted simultaneously with the treatment and the %SHN (χ^2^ = 10.14; df = 1; *p* < 0.01) (Figure 5). Although marginally significant more predators were observed on the second sampling date at farms without flowers compared to the first date, significantly more predators were found at farms with higher landscape complexity without flowers on the second date. Landscape complexity only affected farms without flowers, with significant variation in the slopes between the two sampling dates (estimate = 0.37; std. error = 0.11; z value = 3.18 Pr(>|z|) = 0.001) (Table 2, Figure 4).

For the pitfall traps, as the most common predators were spiders, we only analyzed their abundance; however, it was low (1.01 ± 1.34 for all farms). Between treatments, the mean abundance/trap was, for farms with flowers, 1.11 ± 1.36 and, for farms without flowers, 0.89 ± 1.32. Differences in abundances between treatments were not statistically significant (χ^2^ = 1.08; df = 1; *p* = 0.3) or for %SNH (χ^2^ = 0.9; df = 1; *p* = 0.34) or its interaction (χ^2^ = 0.07; df = 1; *p* = 0.8) (see Figure S1). We found 237 spiders in all sampled fields, distributed in six families: Anyphenidae, Cheiracanthiidae, Lycosidae, Salticidae, Trachelidae, and Linyphiidae, with the last one being the most abundant (*n* = 141).

## 4. Discussion

Habitat diversification strategies can promote biological control [4]; however, these have traditionally been measured without knowledge of their interaction with the surrounding landscape’s characteristics. Studies that have considered both effects are still scarce. For example, in a recent meta-analysis [31], only a small sample size (18 studies were considered) followed the criteria for drawing conclusions. Therefore, many studies that reported no effects of the addition of flower strips without considering the landscape may have overlooked this important aspect. Also, among the many reasons why the provision of flowers may not prove significantly differences could be the fact that many parasitoid species may not be sugar-limited in the field and that the simple and convenient access to honeydew may then hinder the effect of nectar on the fitness of the parasitoids, ultimately affecting the biological control of pests [11,32]. Other species of parasitoids may even use other sugar resources such as guttation [33] or even damaged fruit, for example, from observations in a field of *D. suzukii* with parasitoid *Leptopilina japonica* (P. Abram personal communication). Regardless of the local source of carbohydrates for parasitoids, these may or not be significantly affected by resources at a greater scale (e.g., around farms), depending in part on their ability to move between all these resources and use them. The effect of the surrounding complex landscapes on parasitoid fitness and attraction may therefore reduce local sugar limitations and could eventually change the effect of adding a flower strip (following the hypothesis in [34]). Thus, farms in more simple landscape contexts could benefit greatly by the use of flower strips, if parasitoids are sugar-limited, as the addition of subsidiary resources could allow natural enemies to exploit these easily. However, if other sugar resources are even more available, such as honeydew in an aphid–parasitoid trophic interaction, are present and these satisfy their dietary needs, the addition of flowers would not make any difference (e.g., Ref. [11]). In these cases, the host for oviposition may also provide ample amounts of sugar in the field through their honeydew. In our study, the effect of flowers on the proportion of mummies was greater at farms with flowers, with some modulation by the %SNH, depending on the sampling date. Although the mean aphid abundance was not affected by the presence of flowers or by the sampling date alone, aphid abundance was significantly reduced at farms with flower strips later in the season. The latter could be an effect of the flowers, which may have promoted natural enemy activity early in the season. Thus, buckwheat’s and faba bean’s extra floral nectar here could have enhanced natural enemies’ fitness early in the crop season. Then, a time delay would be expected on the effect of the host, as natural enemy populations need to increase in order to promote a significant reduction in aphid numbers. Our data suggested that aphid parasitism was enhanced by flowers, having a potential early-season effect that ultimately reduced aphid numbers through time. The timing of parasitism has been suggested to be key in controlling aphids [35] and other pests [36,37], highlighting the importance of having nectar and pollen early in the season when pests numbers are still low and can be handled by local natural enemy communities. However, we also observed that aphid mummies that occurred under the absence of flowers were statistically different between sampling dates (Table 2), with fewer numbers initially recorded and their numbers increasing over time, affected as well by the greater %SNH (Figure 2).

The later effect of the landscape in both treatments suggests that key resources may have been present later on in the surrounding landscape, as it significantly increased the proportion of mummies even in the absence of flowers (Table 2). The presence of surrounding native vegetation has been shown to increase the parasitoid activity and parasitism in arable fields [36]. However, after a month of flowering, it is very likely that our flower strips were not supplementing enough nectar, and the parasitoid relied more on the surrounding resources, so we only perceived a stronger effect of the landscape at this later date. Indeed, we found *Raphanus raphanistrum*, *Brassica rapa*, *Capsella bursa-pastori*, and *Lamium amplexicaule* as the main flowering annual plants surrounding the area towards the end of the season.

As proposed by [34], farms in landscapes that are simple to medium in their complexity would in theory benefit more from local management, as we found in our study system early in the season. Although the landscape where these agricultural exploitations occur do not have farms with an SNH greater than 10.8%, which is considered rather low in the literature (e.g., [10]), we still found an increased proportion of mummies during the late part of the sampling period at farms with a higher %SNH. The landscape gradient available in this system may not be enough to test the hypothesis in [34] and therefore fails to provide evidence for the lack of an effect of flower strips when the %SNH is medium to high (e.g., over 20–35% [10]). Within our study constraints, we observed lower per-plant aphid abundances, with greater mummification rates at peak aphid density (15 November) at farms with flower strips of faba beans and buckwheat. Eventually, this effect could be later supplemented by surrounding landscape resources at specific farms showing some complementary effects of the landscape and the local resources. The main parasitoid species detected at our farms, *D. rapae*, has been shown to be positively affected by extra floral nectar, with an increase in survival in laboratory studies (up to 6 more days) compared to a control [38]. On the other hand, buckwheat has been shown to increase the longevity and fecundity of *D. rapae* [39]. We also detected different proportions of fed parasitoids at different sites (unpublished results) for *D. rapae*, suggesting the potential of sugar limitation in the field.

The abundance of generalist predators consisting mainly of Coccinellidae, Lycosidae (Araneae), and Linyphiidae (Araneae) did not change significantly among farms with or without flower strips, except for during the second sampling period, where we found marginally significant more predators at farms without flowers strips and a higher %SNH (Figure 4). At this stage, we cannot suggest that the resource added by these plants deterred these predator on the first sampling date, or it may have just been an effect of the small number of captures. As in previous work, the abundance of ground-dwelling predators in general is low in central Chile in highly intensive agricultural landscapes [40,41]. Also, the time of the year when brassicas are present does not coincide with the higher abundances of Carabids. In the same landscape, for example, [40] found the greatest abundances very late in the season. However, the surrounding landscape seems more important for the abundance of the plant foraging predators we collected on the second sampling date, suggesting that different resources may have been present for predators where greater %SNH values were found surrounding the crops, which increased their abundance. A recent study on brassica crops from southern China suggested that the presence of native forests and grasslands increases spider abundance and species richness [42]. Moreover, these natural enemies have been shown to respond to the landscape diversity and the %SNH in similar Chilean agricultural landscapes associated with apple orchards (Romero, unpublished); however, the landscape gradient was greater (2–73%), which may be necessary to detect measurable differences in predators. Intensely managed landscapes, such as those where most of the vegetable crops are grown in Central Chile, are simpler landscapes with fewer seminatural habitats present [16] and high pesticide input. The flower strips used here had an effect on these parasitoids, which had smaller dispersal rates and therefore may have been more affected by the local management compared to the predators. Further research on coccinellid diets, which are underway, may allow us in the future to identify the specific resources that these insects use. Flower strips may provide resources for certain types of natural enemies and not others [34], so, at least under the studied landscape, flower strips had no effect on predator abundance. Another study carried out in Central Chile suggested that coccinellid abundances (same four common species as observed here) are mostly driven by aphid abundances and not intercropping treatment, which consisted in this particular study of winter cereals with abundant aphids (honeydew) [40]. In Ref. [40], contrary to the results presented here, no relationship with the studied variables or with aphid abundances in one direction or the other was found; however, the potential effect of the landscape was not measured or considered. Therefore, in vegetable crops, it may be necessary to include the abundance of other prey items (e.g., thrips), as aphids may not the most important prey item for this assemblage of predatory species. In a different study, we found a high frequency of intra-guild predation (IGP) between these species [43] as well as Haplothrips (Romero, unpublished) when studying the diet of *Hippodamia variegata*, *Eriopis chilensis*, *Hippodamia convergens*, and *Harmonia axyridis*, which are the four most common species in these landscapes.

For spiders, we did not observe an increase with the presence of flower strips independent of the landscape variable, although adding plant diversity and architectural vegetation complexity may increase spider abundance according to the literature [44]. In the case of spiders, it is not clear whether they act as natural enemies; therefore, they could be responding to other prey items not studied here, potentially to some other predators. The abundance of other prey items should be included in the future, and diet analyses of the predators found here are already underway to identify the missing resources that could help explain these patterns. However, at a more observational level, farms seem to have provided favorable conditions, such as anchoring points for webs and opportunities to use different foraging methods, as suggested by [45], as different hunting guilds were observed at the farms (e.g., space web weavers, sheet web weavers, and other hunters).

## 5. Conclusions

In general, we expect that with a more diverse and structurally more complex plant community, more diverse natural enemy assemblages will occur, which could translate to more effective pest regulation; however, the proportion of mummies was mainly affected by our explanatory variables. In any case, even in very intense vegetable landscapes, the use of broad beans during winter and the sowing of *F. esculentum* in early spring could be effective in reducing aphid populations at brassica farms.

## Figures and Tables

**Table 1:** Landscape cover per farm, geographical coordinates, and management treatments. AWV: area without vegetation (ha); ST: urban settlements (ha); NF: native forest (h); BW: water (ha); AS: arborescent scrub (ha); PL: forest plantation (ha); GR: agricultural grassland (ha); CP: crop field (ha); TBF: total buffer (ha); BSH: natural habitat (ha); PBSH: proportion of natural habitat (ha) (NF + AS); PBW: proportion of water (BW) (ha); % SNH: proportion of seminatural area (PBSH + PBW).

Field	GPS	Treatment	AWV	ST	NF	BW	AS	PL	GR	CP	TBF	BSH	PBSH	PBW	%SNH
Farm 1	35°33′42″ S 71°34′51″ W	with flowers	0	10.4	1.1	0	0.7	0	0	75.1	87.5	1.9	2.2	0	2.2
Farm 2	35°31′03″ S 71°37’29″ W	with flowers	0.5	5.9	0.5	0	0	0	0	118.3	124.9	0	0.5	0	0.5
Farm 3	35°31’37″ S 71°38’40″ W	with flowers	0	0	2.5	0.2	0.1	0.03	0	99.9	102.9	2.7	2.6	0.2	2.8
Farm 4	35°32’42″ S 71°36’25″ W	with flowers	1.09	0	2.1	0	0	0	0	113.2	116.5	2.1	1.8	0	1.8
Farm 5	35°04’41″ S 71°35’59″ W	with flowers	0	15.3	1.1	0	11.4	0	0	87.9	115.9	12.5	10.8	0	10.8
Farm 6	35°33’02″ S 71°38’56″ W	with flowers	0	0.2	3.7	0	0	6.4	0.4	98.7	109.5	3.7	3.4	0	3.4
Farm 7	35°32′54″ S 71°36′21″ W	without flowers	0	0	1.9	0	0	0	0	110.8	112.7	1.9	1.7	0	1.7
Farm 8	35°32′31″ S 71°37′38″ W	without flowers	0	3.8	3.6	0	0	0	0	93.0	100.6	3.6	3.6	0	3.6
Farm 9	35°31′51″ S 71°36′49″ W	without flowers	0.4	3.9	2.3	0	0	0	7.2	113.3	127.3	2.3	1.8	0	1.8
Farm 10	35°33′05″ S 71°39′25″ W	without flowers	0	6.5	0	0	8.4	2.6	0	92.0	109.6	8.4	7.6	0	7.6
Farm 11	35°31’11″ S 71°38’02″ W	without flowers	0	3.3	9.3	0	0	0	0	151.1	163.7	9.3	5.6	0	5.6
Farm 12	35°30’15″ S 71°38’12″ W	without flowers	0	2.9	5.0	0	3.7	0	0	100.5	112.3	8.7	7.8	0	7.8
Farm 13	35°33’07″ S 71°37’44″ W	without flowers	0.3	0.6	7.5	0	0	0	0	82.7	91.2	7.5	8.2	0	8.2
Farm 14	35°32’37″ S 71°37’14″ W	without flowers	0	5.0	0.7	0	0	0	0	100.7	106.5	0.7	0.7	0	0.7

**Table 2:** Response variables from two sampling periods at farms with and without flowers with %SNH as a liner predictor. Type II ANOVA model output after model simplification (see Suppl. Material for all models tested).

Random Effects			Pr (Chisq)	*Df*	χ^2^	
Response: Mean aphids plant/farm						
Std. Dev. 1.66 1.6	Variance 2.77 2.58	Groups sample farm	0.16	1	1.96	%SNH
			0.85	1	0.031	Treatment
			0.12	1	2.41	Date
			3.6 × 10^−07^	1	25.88	% SNH:date
			5.8 × 10^−06^	1	20.53	Date:treatment
Response: Mean prop. mummies plant/farm						
Std. Dev. 2.03 1.35	Variance 4.14 1.82	Groups sample farm	0.71	1	0.13	%SNH
			0.22	1	1.48	Treatment
			1.66 × 10^−4^	1	14.17	Date
			4.83 × 10^−7^	1	25.33	% SNH:date
			0.0479	1	3.91	Date:treatment
Response: Mean abund. total predators plant/farm						
Std. Dev. 0.77	Variance 0.59	Groups farm	0.96	1	0.0016	%SNH
			0.5	1	0.44	Treatment
			0.03	1	4.25	Date
			0.0014	1	10.13	% SNH:date:treatment
	Pr(>|z|) = 0.11	Z = 1.57	SE = 0.10		Estimator −0.15	% SNH:date1:treatment (without flower)
	Pr(>|z|) = 0.001	Z = 3.18	SE = 0.11		Estimator 0.37	% SNH:date2:treatment (without flower)

## Data Availability

Raw data is available upon request to the corresponding author.

## Supplementary Materials

The following supporting information can be downloaded at: https://www.mdpi.com/article/10.3390/insects16030286/s1, Figure S1: Mean number of spiders per pitfall trap ± SE (10 traps per farm) independent of the management treatment (with or without flowers) across the landscape gradient (%SNH in the surrounding 1 km buffer). Blue line represent model prediction, gray area confidence intervals at 95%. Abundance was not statistically different between treatments and %SNH surrounding farms (%SNH: χ^2^ = 0.9038, df = 1, *p* = 0.3418; treatment: χ^2^ = 1.0856, df = 1, *p* = 0.2975); Table S1: Most robust models tested for three response variables, effect on the independent variables, of different random factors, with resulting AIC, residual deviance, χ^2^, Df, and *p* value per model.

## Abbreviations

The following abbreviations are used in this manuscript:

MDPI	Multidisciplinary Digital Publishing Institute
DOAJ	Directory of open access journals
TLA	Three letter acronym
LD	Linear dichroism
