# Supplementary material for: Interaction Effects of Farm-Scale Management of Natural Enemy Resources and the Surrounding Seminatural Habitat on Insect Biological Control
Source: Insects. 2025 Mar 10;16(3):286. doi: 10.3390/insects16030286 (PMC11942830; doi:10.3390/insects16030286)
Supplement: Supplementary file 1 [file insects-16-00286-s001.zip › insects-3494016-supplementary.pdf]

Supplementary material

Table S1. Most robust models tested for three response variables, effect on the independent variables, of different random factors, with resulting AIC, residual deviance, X^2, Df, and *p* value per model.

Independent Variable	Random Factor	AIC	Residual Deviance	X^2	Df	*p* Value
Full model without outlier<br>Response: aphidstot<br>model: glmer.nb(aphidstot~SNH x treatmentxdate)						
SNH	farm + sample	5045.3	1069	2.0190	1	0.1553
Treatment				0.0331	1	0.8556
Date				2.3543	1	0.1249
SNH:treatment				0.5651	1	0.4522
SNH:date				25.861	1	3.669×10^{-7} ***
Treatment:date				20.552	1	5.803×10^{-6} ***
SNH:treatment:date				0.0289	1	0.8649
Selected model without outlier<br>Response: aphidstot<br>Model: glmer.nb(aphidstot~SNH:date + treatment:date + SNH + treatment + date)						
SNH	farm + sample	5041.9	1071	1.9588	1	0.1616
Treatment				0.0305	1	0.8613
Date				2.4129	1	0.1203
SNH:date				25.899	1	3.597×10^{-7} ***
Date:treatment				20.567	1	5.757×10^{-6} ***
Full model with outlier<br>Response: aphidstot<br>Model: glmer.nb(aphidstot~SNHxtreatmentxdate)						
SNH	farm	5184.8	1150	1.1098	1	0.292132
Treatment				0.0192	1	0.889838
Date				10.299	1	0.001331 ***
SNH:treatment				2.5139	1	0.112848
SNH:date				31.502	1	1.992e–08 ***
Treatment:date				19.029	1	1.287e–05 ***
SNH:treatment:date				0.7600	1	0.383337
Selected model with outlier<br>Response: aphidstot<br>Model: glmer.nb(aphidstot~SNH:date + treatment:date + SNH + treatment + date)						
SNH	farm	5183.9	1152	0.8713	1	0.350583
Treatment				0.0185	1	0.891700
Date				9.4731	1	0.002085 **
SNH:date				27.830	1	1.324×10^{-7} ***
Date:treatment				19.012	1	1.299×10^{-5} ***
Full model without outlier<br>Response: tmummies/aphidstot<br>Model: glmer(cbind(tmummies, aphidstot)~SNHxdatextreatment)						
SNH	farm + sample	1953.2	633	0.1912	1	0.6619
Date				13.768	1	0.0002
Treatment				1.5217	1	0.2173
SNH:date				26.341	1	2.861×10^{-7}
SNH:treatment				1.7002	1	0.1922

Date:treatment				3.8955	1	0.0484
SNH:date:treatment				2.7217	1	0.0989
Selected model without outlier<br>Response: tmummies/aphidstot<br>Model: glmer(cbind(tmummies,aphidstot)~SNH:date + treatment:date + SNH + treatment + date)						
SNH	farm + sample	1953.8	635	0.1373	1	0.7109
Treatment				1.4830	1	0.2232
Date				14.177	1	0.0001
SNHdate				25.330	1	4.831×10^{-7}
Date:treatment				3.9098	1	0.048
Full model without outlier<br>Response: predators<br>Model: glmer.nb(predators~SNH + treatment)						
SNH	farm	1089.5	1057	0.0031	1	0.955
Treatment				0.3677	1	0.544
Full model without outlier<br>Response: predators<br>Model: glmer.nb(predators~SNH:date + treatment:date + SNH + treatment +date)						
SNH	farm + sample	1085.1	1072	0.0024	1	0.96066
Treatment				0.4207	1	0.51660
Date				5.2402	1	0.02207 *
SNH:date				3.9594	1	0.04661 *
Date:treatment				0.1087	1	0.74160

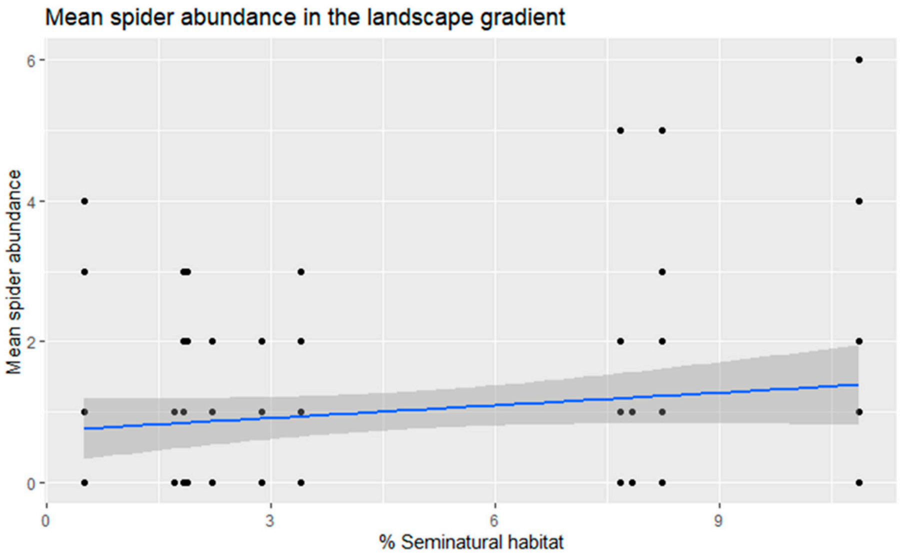

Figure S1. Mean number of spiders per pitfall trap ± SE (10 traps per farm) independent of the management treatment (with or without flowers) across the landscape gradient (%SNH in the surrounding 1 km buffer). Blue line represent model prediction, gray area confidence intervals at 95%. Abundance was not statistically different between treatments and %SNH surrounding farms (%SNH: $X^2 = 0.9038$, df = 1, $p = 0.3418$; treatment: $X^2 = 1.0856$, df = 1, $p = 0.2975$).
